# Gender differences in autonomic and psychological stress responses among educators: a heart rate variability and psychological assessment study

**DOI:** 10.3389/fpsyg.2024.1422709

**Published:** 2024-10-08

**Authors:** Andrea Calderón-García, Estela Álvarez-Gallardo, Pedro Belinchón-deMiguel, Vicente Javier Clemente-Suárez

**Affiliations:** ^1^Department of Pharmacy and Nutrition, Faculty of Biomedical and Health Sciences, Universidad Europea de Madrid, Villaviciosa de Odón, Spain; ^2^Department of Nursing, Faculty of Sport Sciences and Physiotherapy, Universidad Europea de Madrid, Villaviciosa de Odón, Spain; ^3^Faculty of Sports Sciences, Universidad Europea de Madrid, Madrid, Spain; ^4^Research Group in Culture, Education and Society, University of the Coast, Barranquilla, Colombia

**Keywords:** gender differences, stress perception, heart rate variability, educators, autonomic modulation, psychological assessment

## Abstract

**Introduction:**

This study explores the gender differences in psychological stress perception and autonomic modulation among teachers.

**Methods:**

Utilizing heart rate variability (HRV) as a measure of autonomic function and a suite of validated psychological tests, the study examines the discrepancies in stress, anxiety, burnout, and personality traits between male and female educators.

**Results:**

Results indicate that despite higher reported levels of stress and anxiety, women demonstrate a higher HRV, suggesting a stronger parasympathetic response.

**Discussion:**

These findings highlight the complex interplay between psychological stressors and physiological responses, emphasizing the need for gender-specific interventions in stress management within the educational sector. Implications for enhancing educators’ well-being and performance through tailored strategies are discussed.

## 1 Introduction

Stress is a multifaceted organic response designed to address environmental demands, particularly situations perceived as threats, such as psychological, social, or occupational stressors (McEwen, 2000). This triggers a cascade of neurological, psychological, and behavioral responses, primarily mediated by the autonomic nervous system (ANS), with the sympathetic nervous system (SNS) playing a crucial role by escalating its activity in such scenarios (Barman and Yates, 2017). Contemporary social dynamics induce constant exposure to stress that can become chronic, leading to an overstimulation of sympathetic activity and resulting in mental, behavioral, and physical impairments, manifesting in pathologies like anxiety or depression (Clemente-Suárez and Ruisoto-Palomera, 2020).

Current neurobiological advances recognize heart rate variability (HRV) as a reliable indicator for objectively assessing stress, as well as physical and mental health (Kim et al., 2018). HRV exhibits a complex structure, often described as “chaotic,” consisting of oscillations of multiple overlapping frequencies that are interconnected in a nonlinear manner (Lehrer and Gevirtz, 2014). These oscillations represent the activity of homeostatic reflexes, helping to maintain allostatic balance and adapt to environmental demands (Lehrer and Eddie, 2013; Lehrer et al., 2000). HRV reflects the variations in the interval between consecutive heartbeats (R-R Intervals), representing the ANS’s influence over heart rate, showcasing the heart’s ability to adapt to diverse physiological and environmental stimuli, serving as an indirect index of stress (Acharya et al., 2006).

HRV is known to be associated with various effects on the cardiovascular system, the respiratory system, and emotional reactivity (Lehrer, 2013). HRV is modulated through interactions occurring within the pathways of Respiratory Sinus Arrhythmia (RSA) and the baroreflex system (Lehrer, 2013). RSA is the fluctuation of heart rate in synchrony with breathing, modulating heart rate via the vagus nerve, the primary parasympathetic nerve innervating the cardiovascular system (Lehrer, 2013). It is sometimes used as an index of parasympathetic tone and is referred to as “high-frequency heart rate” oscillations (Lehrer et al., 2000). Heart rate accelerates during inhalation and decelerates during exhalation (Lehrer et al., 2000). Consequently, RSA is reduced during periods of stress (Lehrer, 2013). The baroreflex system consists of baroreceptors, which are pressure-sensitive receptors located in large blood vessels such as the carotid artery and the aorta. These baroreceptors play a crucial role in regulating blood pressure and heart rate through feedback mechanisms that respond to mechanical changes in the vascular wall (Lehrer, 2013). The baroreflex system is primarily mediated by the sympathetic nervous system and appears to play a role in regulating vascular tone. These oscillations, known as “low-frequency heart rate” oscillations, have been linked to baroreflex activity (Lehrer et al., 2000). Therefore, it is highly relevant to analyze the different parameters following the guidelines of the Task Force of the European Society of Cardiology and the North American Society of Pacing and Electrophysiology (Malik et al., 1996), which include: minimum heart rate (HRmin); mean heart rate (HRmean); maximum heart rate (HRmax); the root mean square of the successive differences between adjacent normal R-R intervals (RMSSD); the percentage of differences between adjacent normal R-R intervals greater than 50 ms (PNN50); the ratio between low-frequency and high-frequency bands (LF/HF); the low-frequency band in normalized units (LFn); the high-frequency band in normalized units (HFn); and the short-term variability (SD1) and long-term variability (SD2) from the nonlinear spectrum of HRV. The R-R series were analyzed using Kubios HRV software (version 3.0, Biosignal Analysis and Medical Imaging Group, University of Kuopio, Finland).

HRV is controlled by different autonomic pathways (Lehrer and Gevirtz, 2014). The ANS is divided into the sympathetic (SNS), which is activated in response to stress, increasing heart rate and reducing HRV, and the parasympathetic (PNS), which predominates in states of relaxation, increasing HRV (Ortiz-Guzmán et al., 2023). It has been observed that a decrease in HRV is associated with a reduced capacity of the body to manage internal and external stressors. Consistently, higher resting HRV correlates with greater resilience to emotionally stressful situations (Järvelin-Pasanen et al., 2018). Conversely, a low HRV has been established as a marker for various pathologies, including cardiovascular risks, metabolic syndrome, inflammatory processes, cognitive decline, anxiety, depression, and less healthy aging (Žunkovič et al., 2023). The general population appears to live in a state of chronic sympathetic hyperactivity due to continuous stressors, such as occupational tensions, thus increasing the risk of psychophysiological disorders in the medium and long term (Azulay et al., 2022).

It is also pertinent to evaluate self-perception of stress and other factors such as anxiety and Burnout syndrome through validated psychological tests (Redondo-Flórez et al., 2020). Tests measuring stress and work overload, along with HRV, are of particular interest in occupational settings, where work-related stress disorders pose a significant public health challenge (Järvelin-Pasanen et al., 2018). In the educational field, analyzing Burnout and stress in education professionals is crucial, given their high level of exposure to these during the academic year (Agyapong et al., 2022). Previous research has demonstrated significant sympathetic activation in teachers, related to the stressful work environment and exhaustion (Mendoza-Castejon et al., 2020).

Research also indicates gender differences in the reaction and management of stress in complex work environments (Goubet and Chrysikou, 2019). Studies suggest that while women may show higher levels of work stress or anxiety in tests, paradoxically, they exhibit higher HRV values, justified by lower sympathetic activation and a possible parasympathetic dominance at rest (Kim et al., 2018).

Building upon the established premise that gender may influence the autonomic and psychological response to stress, this study aims to delve into the gender-specific autonomic and psychological profiles of teachers. We hypothesize that the chronic stress experienced by educators, particularly in the heightened context of their professional environment, may exhibit distinct patterns of autonomic regulation and psychological impact between male and female teachers. Specifically, we anticipate that these differences may be reflected in varying HRV levels and self-reported measures of stress, anxiety, and burnout, potentially elucidating underlying mechanisms that contribute to gender disparities in stress response within the teaching profession. This analysis is crucial for designing targeted interventions to bolster resilience and well-being among educators.

## 2 Methodology

### 2.1 Design, setting, and participants

To ensure the quality of the article, standard guidelines for high-quality cross-sectional studies were followed (STROBE, 2024). A descriptive cross-sectional study was conducted involving 235 teachers (28.10% (66) male; 71.90% (169) female) from both compulsory education and university-level teaching. A total of 77.40% (183) taught at educational centers in the Community of Madrid (Compulsory and Non-University Higher Education), and 22.60% (Deng et al., 2016) at Spanish Universities (University Education). Participants in this study were teachers from both compulsory education and university-level institutions. The inclusion criteria required that participants be actively employed as educators within the selected institutions during the 2022–2023 academic year. They also needed to provide informed consent and be willing to participate in heart rate variability measurements and complete a series of psychological assessments. Exclusion criteria included having a known cardiovascular disease, chronic psychiatric conditions, or any condition that could interfere with HRV measurements, such as the use of beta-blockers or other heart rate-modifying medications.

Participants were recruited voluntarily. Recruitment was conducted via email invitations sent through institutional mailing lists and announcements during faculty meetings. Teachers who expressed interest were provided with detailed information about the study’s objectives, procedures, and ethical considerations before giving their consent. Participation was entirely voluntary, and teachers could withdraw from the study at any time without consequences. The sample size was calculated using a power analysis based on the expected differences in HRV parameters between male and female participants. We aimed to detect a medium effect size (*d* = 0.5) with 80% power and a significance level of 0.05. The calculated minimum required sample size was 200 participants. However, to account for potential dropouts and ensure robustness, we aimed for a larger sample size. Ultimately, 235 teachers (66 male and 169 female) participated in the study.

Data were collected at the beginning of the 2022–2023 school year. Subsequently, the data were anonymized and stripped of any information that could identify the participants. Inclusion required obtaining informed consent, respecting the bioethical principles of the most updated version of the Declaration of Helsinki (Médica, 2013). The study was approved by the European University of Madrid, Spain, Ethics Committee under code CIPI/22.178.

### 2.2 Instruments

The autonomic modulation of participants was analyzed through the study of heart rate variability (HRV). HRV was monitored using a Polar V800 heart rate monitor (Polar, Kempele, Finland), which participants wore for a minimum of 6 min while seated. The analyzed parameters followed the guidelines of the Task Force of the European Society of Cardiology and the North American Society of Pacing and Electrophysiology (Agyapong et al., 2022), and included: minimum heart rate (HRmin); mean heart rate (HRmean); maximum heart rate (HRmax); the square root of the sum of the squares of differences between normal adjacent R-R intervals (RMSSD); the percentage of differences between normal adjacent R-R intervals greater than 50 ms (PNN50); ratio between low and high frequency bands (LF/HF); the low-frequency band in normalized units (LFn); the high-frequency band in normalized units (HFn); and the sensitivity of the short-term variability (SD1) and long-term variability (SD2) from the non-linear spectrum of HRV. The R-R series were analyzed using Kubios HRV software (version 3.0, Biosignal Analysis and Medical Imaging Group, University of Kuopio, Finland). To ensure the accuracy of HRV measurements, all data were carefully screened for artifacts. Artifacts, defined as non-physiological irregularities in the R-R intervals (e.g., due to movement or poor electrode contact), were identified and corrected using the artifact detection algorithms integrated into the Kubios HRV software (version 3.0, Biosignal Analysis and Medical Imaging Group, University of Kuopio, Finland). These algorithms apply threshold-based detection and interpolation techniques to minimize the impact of artifacts on HRV analysis. Only recordings with less than 5% corrected data were included in the final analysis to maintain data integrity.

Additionally, a total of 169 participants (71.92%) completed a series of psychological questionnaires in their Spanish-validated versions. The tests were autonomously completed on mobile devices and included:

- Perceived Stress Scale (PSS) (Mendoza-Castejon et al., 2020), in its short Spanish-validated version, assessing the level of stress perceived over the last month through 4 items. It presents a Cronbach’s alpha coefficient of 0.799.
- Zung’s Self-rating Depression Scale (Goubet and Chrysikou, 2019), which consists of 20 items related to depression: 8 items associated with cognitive aspects, 8 with somatic aspects, 2 related to mood, and the last 2 with psychomotor symptoms. It presents a Cronbach’s alpha coefficient of 0.269.
- Anxiety levels by the short version of the State–Trait Anxiety Inventory (STAI). The short form of the STAI has been validated for reliability and accuracy in capturing both state anxiety, which refers to the temporary and situation-specific anxiety, and trait anxiety, which indicates a general predisposition to experience anxiety. Following the validation methodology outlined by Marteau and Bekker (Iwasa et al., 1970), we administered the short STAI to ensure that our measures were both efficient and psychometrically sound. The short version demonstrated strong internal consistency, with a Cronbach’s alpha of 0.82 for the state anxiety scale and 0.90 for the trait anxiety scale. Additionally, the test–retest reliability was found to be 0.76 for state anxiety and 0.80 for trait anxiety.
- Maslach Burnout Inventory (MBI) (STROBE, 2024) in the Spanish-validated version (Médica, 2013), which assesses the presence of Burnout Syndrome. It consists of 22 statements measuring the frequency and intensity with which a professional suffers from burnout, evaluating 3 dimensions: emotional exhaustion (EE), depersonalization (DP), and personal accomplishment (PA). It presents a Cronbach’s alpha coefficient of 0.632.
- BIGFIVE (Solis et al., 1983), with 10 items that assess five personality traits: extraversion, agreeableness, conscientiousness, neuroticism, and openness to experience. It presents a Cronbach’s alpha coefficient of 0.770.
- Acceptance and Action Questionnaire-II (AAQ-II) (Zung, 1965) in its Spanish version to measure experiential avoidance and psychological inflexibility using a 7-item construct with a 7-point Likert scale, where 0 means never true and 7 means always true. It presents a Cronbach’s alpha coefficient of 0.907.
- The Three-Item Loneliness Scale (TIL Scale) from UCLA (University of California at Los Angeles), Spanish-validated (Iwasa et al., 1970), widely used for evaluating the feeling of loneliness. It consists of 3 items answered on a 3-point Likert scale (1 means never and 3 means often). It presents a Cronbach’s alpha coefficient of 0.760.

Finally, all participants were evaluated for “Teacher Satisfaction” using a 0 to 10 Likert scale, where 10 signifies complete satisfaction; and self-perceived “Teacher Stress” using another Likert-type scale from 0 to 10, where 10 signifies severe stress.

### 2.3 Procedures

Heart rate variability (HRV) was recorded using a Polar V800 heart rate monitor, which participants wore for at least 6 min while seated. Prior to the measurement, the teachers were instructed on the importance of remaining at rest and calm during the monitoring, which was conducted in a single session at the beginning of the school year. During the recording, participants maintained natural breathing without the use of biofeedback methods and followed their usual breakfast routine.

Each session was conducted individually during the teachers’ workday in a designated room. The data collection order was organized based on the availability of the teachers over three days at each school. Both male and female participants were subjected to identical conditions, with no gender differences in the procedure.

### 2.4 Statistical procedures

To analyze the data, descriptive statistics, including means and standard deviations, were calculated for all variables using SPSS software (version 24, IBM, Chicago, IL, USA). One-way analysis of variance (ANOVA) was employed to assess differences in heart rate variability parameters and psychological test scores (including the Perceived Stress Scale, State–Trait Anxiety Inventory, and Maslach Burnout Inventory) between male and female participants. Significance was set at *p* ≤ 0.05. Effect sizes for significant results were estimated using Cohen’s d to provide insight into the practical relevance of the findings. In addition, multiple regression analyses were conducted to examine the combined influence of gender, HRV metrics, and psychological test scores on perceived stress levels.

## 3 Results

Table 1 presents the characterization of the sample. The average job satisfaction level was 8.09 ± 1.27 on a 0 to 10 scale, with no significant differences found between genders. However, differences were found in the self-perception of stress levels, being 7.55 ± 1.98 in women compared to 6.73 ± 2.18 in men (*p* = 0.012).

**Table 1:** Characteristics of survey respondents.

		Male	Female	Total	*p*-value
		66 (28.10%)	169 (71.90%)	235 (100%)	
Age (years)	Mean (SD)	40.94 (8.26)	42.66 (9.18)	42.18 (8.95)	*F* = 0.688 *p* = 0.166
Age group	<p50 (43 years)	36 (54.55%)	81 (47.92%)	117 (49.80%)	χ^2^ = 0.831 *p* = 0.386
	≥P50 (43 years)	30 (45.45%)	88 (52.08%)	118 (50.20%)	
Teaching level	Early Childhood and Primary Education	22 (33.30%)	82 (48.52%)	104 (44.20%)	χ^2^ = 8.055 *p* = 0.018*
	Secondary Education/Baccalaureate/Vocational Training	31 (46.97%)	47 (27.81%)	78 (33.20%)	
	University Teaching	13 (19.70%)	40 (23.67%)	53 (22.60%)	
Workplace	Concerted center	31 (46.97%)	77 (45.56%)	108 (46.00%)	χ^2^ = 0.038 *p* = 0.846
	Private center	35 (53.03%)	92 (54.44%)	127 (54.00%)	
Knowledge area^1^	Health science	21 (51.20%)	44 (44.00%)	65 (46.10%)	χ^2^ = 17.529 *p* = 0.002
	Science	3 (7.30%)	24 (24.00%)	27 (19.10%)	
	Engineering and architecture	5 (12.20%)	0.00% (0)	5 (3.50%)	
	Social and Legal Sciences	5 (12.20%)	9 (9.90%)	14 (9.90%)	
	Arts and Humanities	7 (17.10%)	23 (23.00%)	30 (21.30%)	
Teaching experience (years)	Mean (SD)	10.77 (8.13)	13.66 (9.98)	12.86 (9.57)	*F* = 4,61 *p* = 0.076
Teaching satisfaction (0–10)	Mean (SD)	8.31 (1.07)	8.01 (1.34)	8.09 (1.27)	*F* = 0.102 *p* = 0.124
Teaching stress (0–10)	Mean (SD)	6.73 (2.18)	7.55 (1.98)	7.32 (2.07)	*U* = 2252.5 *P* = 0.012*

SD, Standard Deviation. *Significant differences *p* < 0.05. Chi-squared test for the comparison of prevalences. Mean comparisons by Student’s *t*-test or Mann–Whitney *U* test depending on whether they follow a normal distribution. ^1^Fisher’s exact test applied because more than 20% of the frequencies have a sample size < 5.

Initially, gender differences in the scores obtained in the psychological tests were evaluated (Table 2). Significant differences were found in the PSS, with females scoring higher, indicating greater perceived stress in the last month (6.99 ± 3.22 vs. 5.88 ± 3.29 *p* = 0.048). There were also differences in the STAI, with females again scoring higher (15.36 ± 4.19 vs. 13.32 ± 4.57 *p* = 0.012), interpreted as experiencing a current emotional state of stress-induced anxiety. Lastly, within the BIGFIVE, differences between genders were noted in the subscale relating to Neuroticism levels, with women scoring higher (5.98 ± 2.13 vs. 5.02 ± 1.94 *p* = 0.007). Although no significant differences were found in the other tests between genders, in all those related to stress, anxiety, or work exhaustion, women scored modestly higher when assessing perceptions of stress, anxiety, or exhaustion (ZUNG, MBI AE, AAQ-II, and TIL Scale UCLA).

**Table 2:** Gender differences in the results obtained in the psychological tests.

Test					Male	Female	Total	*p*-value
PSS	X (SD)				5.88 (3.29)	6.99 (3.22)	6.68 (3.27)	*F* = 0.024 *p* = 0.048*
Zung	X (SD)				44.53 (4.05)	44.90 (4.71)	44.80 (4.53)	*F* = 1.044 *p* = 0.618
	Categories N (%)	Normal range			44 (93.60%)	106 (85.50%)	0 (0%)	χ^2^ = 2.093 *p* = 0.846
		Slightly depressed			3 (6.40%)	18 (14.50%)	0 (0%)	
		Moderately depressed			0 (0%)	0 (0%)	0 (0%)	
		Severely depressed			0 (0%)	0 (0%)	0 (0%)	
MBI	Subescales X (SD)	BEE. Emotional exhaustion	X (SD)		22.35 (11.95)	24.41 (10.75)	23.84 (10.90)	*F* = 0.304 *p* = 0.279
			Categories	Low	16 (23.20%)	34 (18.30%)	50 (19.60%)	χ^2^ = 1.194 *p* = 0.550
				Moderate	34 (49.30%)	105 (56.50%)	139 (54.50%)	
				High	19 (27.50%)	47 (25.30%)	66 (25.90%)	
		BD. Depersonalizacion	X (SD)		4.16 (3.05)	3.85 (3.86)	3.94 (3.64)	*F* = 1.610 *p* = 0.579
			Categories	Low	38 (55.10%)	107 (57.50%)	145 (56.90%)	χ^2^ = 0.301 *p* = 0.860
				Moderate	30 (43.50%)	75 (40.30%)	105 (41.20%)	
				High	1 (1.40%)	4 (2.20%)	5 (2.00%)	
		BPD. Personal accomplishment	X (SD)		32.63 (4.11)	31.67 (5.62)	31.93 (5.97)	*F* = 7.293 *p* = 0.285
			Categorías	Low	18 (26.10%)	63 (33.90%)	81 (31.80%)	χ^2^ = 2.681 *p* = 0.262
				Moderate	47 (30.70%)	106 (57.00%)	153 (60.00%)	
				High	4 (19.00%)	17 (9.10%)	21 (8.20%)	
STAI (A/E)	X (SD)				13.32 (4.57)	15.36 (4.19)	14.82 (4.37)	*F* = 1.289 *p* = 0.012*
BIG FIVE	Subscales X (SD)	Extraversion			5.83 (1.88)	5.46 (1.68)	5.56 (1.74)	*F* = 0.502 *p* = 0.233
		Agreeableness			7.13 (1.90)	7.08 (1.63)	7.09 (1.75)	*F* = 1.890 *p* = 0.887
		Conscientiousness			8.10 (1.55)	8.27 (1.47)	8.23 (1.49)	*F* = 0.399 *p* = 0.514
		Neuroticism			5.02 (1.94)	5.98 (2.13)	5,71 (2.09)	*F* = 0.205 *p* = 0.007*
		Openness to experience.			7.06 (2.06)	8.03 (1.64)	7.91 (1.77)	*F* = 4.546 *p* = 0.201
AAQ-II	X (SD)				19.27 (8.37)	19.65 (9.99)	19.54 (9.46)	*F* = 1.720 *p* = 0.803
TIL Scale UCLA	X (SD)				4.58 (1.66)	4.65 (1.77)	4.63 (1.74)	*F* = 0.272 *p* = 0.830

X, mean; SD, Standard Deviation. *Significant differences *p* < 0.05. Chi-square test used for comparison of prevalences. Mean comparisons using Student’s *T*-test or Mann–Whitney *U* test depending on whether they follow a normal distribution. ^^1^Fisher’s exact test applied because more than 20% of the frequencies have a sample size < 5. PSS, Perceived Stress Scale; Zung, Self-rating Depression Scale; STAI, State–Trait Anxiety Inventory Questionnaire (A-A: State-Anxiety); MBI, Maslach Burnout Inventory; AAQ-II, Acceptance and Action Questionnaire-II; TIL Scale UCLA, The Three-Item Loneliness Scale.

Secondly, differences in HRV parameters and autonomic modulation between genders were examined (Table 3). Significant differences were found between genders in average, minimum, and maximum heart rate, with all being significantly higher in females (average HR: 77.67 ± 12.0 vs. 69.51 ± 11.27 *p* < 0.001; minimum HR: 63.74 ± 13.36 vs. 59.24 ± 9.58 *p* = 0.001; and maximum HR: 104.25 ± 36.49 vs. 86.23 ± 15.20 *p* < 0.001*).

**Table 3:** Gender differences in heart rate variability (HRV).

	Male	Female	Total	*p*-value
Media HR	69.51 (11.27)	77.67 (12.00)	75.35 (12.34)	*U* = 3462.5 *P* < 0.001*
Min HR	59.24 (9.58)	63.74 (13.36)	62,47 (12.55)	*U* = 3927.50 *P* = 0.001*
Max HR	86.23 (15.20)	104.25 (36.49)	99.17 (32.89)	*U* = 3550.5 *P* < 0.001*
RMSSD	43.86 (24.72)	56.57 (48.38)	52.98 (43.37)	*F* = 10.30 *P* = 0.043*
pNN50	18.76 (16.98)	20.04 (20.01)	19.68 (19.18)	*F* = 1.652 *p* = 0.623
LF	76.88 (11.78)	64.59 (16.83)	68.06 (16.51)	*U* = 3126.5 *P* < 0.001*
HF	23.08 (11.77)	34.14 (15.67)	31.02 (15.47)	*U* = 3256.0 *P* < 0.001*
Ratio LF. HF	4.87 (3.57)	2.78 (2.57)	3.34 (3.03)	*U* = 3124.5 *P* < 0.001*
SD1	31.06 (17.51)	40.00 (34.30)	37.47 (30.74)	*F* = 10.349 *p* = 0.045*
SD2	68.77 (28.20)	71.62 (47.51)	70.81 (42.88)	*F* = 45.333 *p* = 0.478

X, mean; SD, Standard Deviation. HR, Heart rate; LF, Low frequency; HF, High frequency; RMSSD: Standard deviation of successive absolute differences of R-R intervals; pNN50: percentage of adjacent intervals varying by more than 50 ms; SD, standard deviation of R-R intervals. * Significant differences *p* < 0.05. Mean comparisons using Student’s *T*-test or Mann–Whitney *U* test depending on whether they follow a normal distribution.

Significant differences were also found in RMSSD, again higher in females (56.57 ± 48.38 vs. 43.86 ± 24.72 *p* = 0.043), indicating greater parasympathetic activity. No significant findings were reported for pNN50, but higher results were again observed in the female sample. The LF/HF ratio was higher in males (4.87 ± 3.57 vs. 2.75 ± 2.57 *p* < 0.001).

The regression analysis showed that higher RMSSD values, which indicate greater parasympathetic activity, were significantly associated with lower reported stress levels (*β* = −0.25, *p* < 0.05). Gender was also a significant predictor, with female participants reporting higher stress levels than their male counterparts (*β* = 0.30, *p* < 0.01). Additionally, higher scores on the State–Trait Anxiety Inventory were positively associated with increased perceived stress (*β* = 0.40, *p* < 0.001). The overall model was significant [*F*(3, 231) = 15.67, *p* < 0.001], explaining approximately 22% of the variance in perceived stress levels (adjusted R^2^ = 0.22). These results suggest that autonomic markers like HRV, along with gender and anxiety levels, play a substantial role in shaping educators’ stress perceptions, underscoring the complex interplay between physiological and psychological factors.

## 4 Discussion

This study was undertaken with the aim of analyzing gender differences in the autonomic and psychological profile of educators. The hypothesis posited that female teachers would exhibit higher levels of stress and a distinctive psychological profile compared to their male counterparts. Through the comprehensive analysis of heart rate variability (HRV) and psychological questionnaires, the research sought to elucidate the intricate interplay between gender, stress, and occupational wellbeing among teachers. The findings of the study indeed support the initial hypothesis, revealing significant gender-based disparities in stress perception, anxiety, and autonomic regulation within the teaching profession.

Through HRV analysis, this study first examined the results of psychological tests with a focus on gender differences. It found that women scored significantly higher in areas of stress (PSS), state anxiety (STAI), and neuroticism (BIGFIVE). Moreover, women generally scored higher on questionnaires associated with emotional exhaustion and stress. These findings are consistent with prior literature that reflects a clear influence of gender on the perception of stress, assessed with the same tools, particularly in the labor context and the teaching profession (Malik et al., 1996; Maslach and Jackson, 1981; Seisdedos, 1997). Women perceive themselves as 18.88% more stressed than men, as indicated in our study (men: 5.88 ± 3.29 vs. women: 6.99 ± 3.22, *p* = 0.048). Similar findings are reported in other studies, such as one involving 470 Spanish university teachers, where women exhibited 12.49% higher perceived than men stress (PSS-14) (22.15 ± 4.40 vs. 19.69 ± 3.61 *p* < 0.0001) (Malik et al., 1996). Another study with 524 higher education teachers in Portugal revealed that women rated Perceived Stress (PSS) 10.92% higher than men (1.93 ± 0.70 vs. 1.74 ± 0.73, *p* < 0.001) (Maslach and Jackson, 1981). Similarly, a study involving 427 Primary and Secondary Education Spanish Teachers found that perceived stress was higher in women (PSS-14) (*p* < 0.05, *d* = −0.30) (Maslach and Jackson, 1981).

When assessing State Anxiety (STAI), it was observed that women scored higher, indicating an association with elevated stress-induced anxiety and potentially poorer adaptation of the Autonomic Nervous System (ANS) in emotionally complex situations. Consistent findings in the literature reveal that women frequently report heightened anxiety and work-related tension during challenging periods, particularly evident in the teaching field, albeit relatively scarce among teachers (Ortet et al., 2017).

This observation aligns with the significant difference in scores on the neuroticism subscale of the BIGFIVE, where women scored markedly higher than men (women: 5.98 ± 2.13 vs. men: 5.02 ± 1.94, *p* = 0.007). This result is supported by prior evidence suggesting a link between higher emotional instability, job exhaustion, and the perceived higher levels of anxiety and stress (Bond et al., 2011). Similar findings were reported in a study with university teachers, revealing a significant difference in the Neuroticism subscale (women: 5.53 ± 1.97 vs. men: 4.77 ± 1.96, *p* = 0.004) (Malik et al., 1996). However, there is a lack of data regarding neuroticism in school teachers. Notably, when compared to other adult populations, teachers’ neuroticism scores appear notably higher, as indicated by a meta-analysis encompassing diverse adult samples worldwide, with average values ranging from 2.05 to 3.21 (Pedroso-Chaparro et al., 2022).

The differentiation is less evident when examining work exhaustion or burnout, with age and years of teaching experience likely being more pertinent factors (Seisdedos, 1997). Contrary to the present study, most literature either finds no clear relationship or concludes that women tend to report higher levels of burnout, particularly when assessed by the Emotional Exhaustion Scale (BEE) (Ingles et al., 2019; Teles et al., 2020). In a study of Portuguese teachers by Teles R (Seisdedos, 1997), Emotional Exhaustion (BEE) evaluated by the Maslach Burnout Inventory (MBI) was 10.83% higher in women than in men (3.07 ± 1.33 vs. 2.77 ± 1.32, *p* < 0.001), comparable to our study with a difference of 9.23% (24.41 ± 10.75 vs. 22.35 ± 11.95, *p* = 0.279), though not reaching statistical significance in this case. Redondo-Flórez’s study with university teachers (Malik et al., 1996) similarly found significance only in the Emotional Exhaustion (BEE) subscale, reporting scores 26.88% higher in women (20.86 ± 9.51 vs. 16.44 ± 9.12, *p* < 0.0001), potentially attributed to the higher demand typically faced by university professors. Finally, Aparisi’s study of Spanish school teachers (Maslach and Jackson, 1981) also achieved significance only in emotional exhaustion (*p* < 0.05, *d* = −0.39). It is noteworthy that the levels of burnout in this study (25.90%) are significantly higher than those reported in the consulted literature (with a prevalence of 5.80–16.00%) (Forcella et al., 2024; Schneider et al., 2023). This is a considerable point, especially considering that the data were collected at the beginning of the school year, and tend to worsen as the course progresses (Stephan et al., 2020).

Lastly, the ZUNG, AAQ-II, and UCLA questionnaires, related to depression, psychological inflexibility, and loneliness, respectively, did not find gender differentiation. These gender divergences in the labor and/or teaching environment have led to multiple theories by various authors that point to differences in the strategies employed in coping with stressful situations between men and women (Malik et al., 1996). Indeed, when correlating the results of the psychological tests with HRV, contrary to expectations, women generally reflect greater HRV, which means they have greater parasympathetic activity and lower sympathetic activity than men.

Individuals with more stress in their daily routine show lower values in the RR interval and in the RMSSD value, and higher in the LF/HF ratio, which assesses low parasympathetic activity (Guidetti et al., n.d.). These results are also observed in individuals with chronic fatigue, metabolic syndrome, or various metabolic and psychophysiological alterations (Seibt and Kreuzfeld, 2021; Marić et al., 2020; García-Real et al., 2024). Such data are particularly striking in teachers, in whom HRV (assessed by RMSSD and LF/HF) decreases throughout the school year due to increased mental exhaustion, corroborated by higher levels of morning cortisol and burnout (Marić et al., 2022). In fact, differences are found in HRV parameters between teaching days and days off for teachers (Dimitriev and Saperova, 2015).

Most of the mentioned research does not differentiate between genders, although the literature finds relevant differences both in HRV and psychological data (Azulay et al., 2022; Ma et al., 2017). This study stands out both for the analysis of gender differences and for the apparent contradictions in its results, already elucidated in previous studies. It is observed that women obtain results in psychological tests indicative of higher personal and labor stress, or more propensity to episodes of anxiety or neuroticism. However, when analyzing HRV parameters, they reflect contradictory results, as they have better cardiac autonomic modulation in women, reflected in higher HRV and parasympathetic activity, higher RMSSD, and lower LF/HF ratio, among others.

The RMSSD value is interpreted as lower and more regular HRV, meaning that the women participants in this study showed greater physiological resilience to stress. LF is a measure of PNS activity, while HF reflects SNS and vagal nerve activity. Therefore, women had lower LF/HF ratios, a reliable estimator of autonomic balance at rest, because it reflects SNS activity (Lehrer and Eddie, 2013). This result suggests that male participants had higher overall SNS activity, especially during the stressful periods of the day. Similar data are found in previous research, such as that conducted on 695 healthy subjects, even after controlling for confounding factors such as age or physical activity (MacIorowska et al., 2020). This study, conducted on 695 subjects spanning different age groups, revealed significant differences between sexes in all measured HRV parameters. Values associated with parasympathetic activity were higher in women for SDNN, RMSSD, pNN50, and HF, while the remaining parameters (LF, LF/HF) were lower.

It is noteworthy to mention a study by Voss et al. (2015), which involved 1,124 men and 782 women. The findings were consistent with our study, demonstrating sex-based differences across various age groups. For instance, RMSSD was higher in women in both young adults (36.5 ± 20.1 vs. 34.0 ± 18.3) and older adults (22.0 ± 13.2 vs. 20.5 ± 11.0), with differences less pronounced than in our study (women: 56.57 ± 48.28 vs. men: 43.86 ± 24.72). Similar patterns were observed in the LF/HF ratio for young adults (women: 2.09 ± 2.05 vs. men: 3.33 ± 3.47) and older adults (women: 2.75 ± 2.93 vs. men: 4.29 ± 4.06), indicative of greater vagal tone in women, consistent with our study (women: 2.78 ± 2.57 vs. men: 4.87 ± 3.57).

Additionally, Kuang et al. (2019) comparing 182 healthy individuals by sex found higher parasympathetic activity in women, represented by greater RMSSD (39.31 ± 17.60 vs. 35.00 ± 19.37) and lower LF/HF (0.97 ± 0.73 vs. 1.80 ± 2.64). Another study by Abhishekh et al. (2013) on 189 healthy participants observed higher LF values in men (46.61 ± 15.17 vs. 40.41 ± 14.95) and lower HF values (42.47 ± 15.3 vs. 45.86 ± 16.4), mirroring our findings. Finally, an investigation by Agelink et al. (2001) involving 309 healthy subjects at rest concluded that women exhibited a lower average LF power (2.85 ± 0.48 vs. 3.03 ± 0.44 msec^2^) and a lower average LF/HF ratio (2.13 ± 2.12 vs. 2.50 ± 2.15 msec^2^) compared to men.

On the contrary, a study on 417 European and American participants (Sammito and Böckelmann, 2016) reported higher HF values in women, signifying greater parasympathetic nervous system activity (men: 22.40 ± 19.51 vs. women: 23.47 ± 22.14), consistent with our study (men: 23.08 ± 11.71 vs. women: 34.14 ± 15.67). Another meta-analysis that included 172 studies concluded again that women showed higher resting HRV than men, translated into a greater parasympathetic response even in difficult or stressful situations (Ma et al., 2017). Similarly, previous studies show that women report feeling negative emotions more intensely, such as stress or anxiety, a reason that could explain why in most psychological tests they achieve (Voss et al., 2015).

### 4.1 Limitations of the study

The current study’s cross-sectional design presents inherent limitations, particularly in establishing causality. Increasing the sample size is a critical next step to improve the statistical power and to confirm the reproducibility of the findings. Moreover, the potential influence of confounding factors, such as physical activity, socio-economic status, and educational environment, were not comprehensively controlled and could have introduced bias. Future research should aim to include these variables for a more nuanced understanding. Additionally, the data collection at the beginning of the academic year may not accurately reflect stress levels and HRV fluctuations throughout the entire year. A longitudinal approach, capturing data at multiple points, would offer a richer, dynamic view of the stress response and its physiological correlates over time.

### 4.2 Future research directions

The intriguing findings on gender differences open several avenues for future investigation. Longitudinal studies should be conducted to examine the progression of stress and burnout over an academic year and to assess the efficacy of targeted interventions. Exploring the interplay between psychological factors and physiological markers of stress in more diverse educational settings can also provide a broader perspective. Additionally, research into the effectiveness of specific coping strategies and their influence on HRV and psychological wellbeing among teachers can yield valuable insights. Investigating the role of emotional intelligence and social support networks in mitigating the impact of occupational stress on teachers could further contribute to developing comprehensive wellness programs. The potential long-term effects of chronic stress on educators’ health and career longevity also warrant further examination.

### 4.3 Practical applications

The significant gender differences in psychological stress responses and autonomic modulation identified in this study underscore the need for gender-specific interventions in educational settings. Schools and universities could consider tailored stress-reduction programs, which might include mindfulness training, regular physical activity, and professional psychological support. Furthermore, regular screening for stress and burnout could be instituted as part of the educators’ professional development programs, helping to identify those at risk and to provide early intervention.

## 5 Conclusion

In conclusion, this study has shed light on the gender-specific psychological and autonomic profiles of teachers, revealing significant differences in perceived stress, anxiety, and autonomic cardiac modulation. Women were found to score higher on stress, state anxiety, and neuroticism, which aligns with previous literature indicating a stronger perceived stress response in females, particularly in the educational context. Paradoxically, despite higher stress and anxiety scores, women also exhibited higher heart rate variability (HRV), suggesting a more robust parasympathetic response, typically associated with better stress resilience.

The data indicate that while women report higher levels of stress and emotional exhaustion, they may also possess a more active parasympathetic nervous system, which could confer protective cardiovascular benefits. This finding points to the complexity of stress responses and the need for nuanced approaches to stress management in the teaching profession. Ultimately, the study emphasizes the importance of recognizing and addressing gender-specific stress and burnout in educators. By applying these insights, educational institutions can better support their staff through targeted interventions, potentially enhancing teacher well-being and effectiveness in the crucial role they play in shaping future generations.

## Data Availability

The original contributions presented in the study are included in the article/supplementary material, further inquiries can be directed to the corresponding author.
