# Correction: Gender differences in autonomic and psychological stress responses among educators: a heart rate variability and psychological assessment study

**DOI:** 10.3389/fpsyg.2026.1821024

**Published:** 2026-04-15

**Authors:** Andrea Calderón-García, Estela Alvarez-Gallardo, Pedro Belinchón-deMiguel, Vicente Javier Clemente-Suárez

**Affiliations:** 1 Department of Pharmacy and Nutrition, Faculty of Biomedical and Health Sciences, Universidad Europea de Madrid, Villaviciosa de Odón, Spain; 2 Department of Nursing, Faculty of Sport Sciences and Physiotherapy, Universidad Europea de Madrid, Villaviciosa de Odón, Spain; 3 Faculty of Sports Sciences, Universidad Europea de Madrid, Madrid, Spain; 4 Research Group in Culture, Education and Society, University of the Coast, Barranquilla, Colombia

**Keywords:** gender differences, stress perception, heart rate variability, educators, autonomic modulation, psychological assessment

The Ethics statement was erroneously given as: “The studies involving human/animal participants were reviewed and approved by the European University of Madrid, Spain, ethics Committee under code CIPI/213006.55.”

The correct Ethics statement is: “The studies involving human participants were reviewed and approved by the European University of Madrid, Spain, Ethics Committee under code CIPI/22.178.”

A correction has been made to section 2 Methodology, sub-section *2.1 Design, setting, and participants*, last paragraph:

“The study was approved by the European University of Madrid, Spain, Ethics Committee under code CIPI/22.178.”

The original version of this article has been updated.

